# On the effectiveness of a contrastive cascade graph learning framework: The power of synthetic cascade data

**DOI:** 10.1371/journal.pone.0293032

**Published:** 2023-10-16

**Authors:** Daiki Suzuki, Sho Tsugawa, Keiichiro Tsukamoto, Shintaro Igari

**Affiliations:** 1 Graduate School of Engineering Information and Systems, University of Tsukuba, Tsukuba, Ibaraki, Japan; 2 Institute of Systems and Information Engineering, University of Tsukuba, Tsukuba, Ibaraki, Japan; 3 KADOKAWA Connected Inc., Chiyoda-ku, Tokyo, Japan; University of Mauritius, MAURITIUS

## Abstract

Analyzing the dynamics of information diffusion cascades and accurately predicting their behavior holds significant importance in various applications. In this paper, we concentrate specifically on a recently introduced contrastive cascade graph learning framework, for the task of predicting cascade popularity. This framework follows a pre-training and fine-tuning paradigm to address cascade prediction tasks. In a previous study, the transferability of pre-trained models within the contrastive cascade graph learning framework was examined solely between two social media datasets. However, in our present study, we comprehensively evaluate the transferability of pre-trained models across 13 real datasets and six synthetic datasets. We construct several pre-trained models using real cascades and synthetic cascades generated by the independent cascade model and the Profile model. Then, we fine-tune these pre-trained models on real cascade datasets and evaluate their prediction accuracy based on the mean squared logarithmic error. The main findings derived from our results are as follows. (1) The pre-trained models exhibit transferability across diverse types of real datasets in different domains, encompassing different languages, social media platforms, and diffusion time scales. (2) Synthetic cascade data prove effective for pre-training purposes. The pre-trained models constructed with synthetic cascade data demonstrate comparable effectiveness to those constructed using real data. (3) Synthetic cascade data prove beneficial for fine-tuning the contrastive cascade graph learning models and training other state-of-the-art popularity prediction models. Models trained using a combination of real and synthetic cascades yield significantly lower mean squared logarithmic error compared to those trained solely on real cascades. Our findings affirm the effectiveness of synthetic cascade data in enhancing the accuracy of cascade popularity prediction.

## Introduction

Analyzing the dynamics of information diffusion cascades and accurately predicting their behavior holds significant importance in various applications, such as viral marketing [1], information recommendation [2], and fake-news detection [3]. The dissemination of information through functionalities such as “like” and “retweet” on social media platforms creates cascades of information diffusion [4]. Predicting the future popularity of these cascades at their early stages has been a crucial research topic for the aforementioned applications [5]. Additionally, predicting other aspects of information cascades, such as outbreak detection [6–8] and susceptibility estimation [9–11], has garnered considerable attention from researchers [5, 12–18].

Three main approaches exist for predicting the future dynamics of information diffusion cascades: feature-based [17, 18], model-based [15, 16], and deep-learning-based [5, 12–14]. Feature-based methods employ various cascade characteristics, such as user participation times, structural properties of the cascade graph, and content information, to predict cascade dynamics [17, 18]. Model-based methods utilize mathematical models to capture the dynamics of information diffusion cascades [15, 16]. Deep-learning-based methods leverage neural networks to learn latent features of cascades [5, 12–14].

Of these three approaches, the deep-learning-based one has been considered as the most promising owing to its high accuracy [4]. Several studies [7, 19] have demonstrated that deep-learning-based methods outperform feature-based and model-based methods in terms of prediction accuracy for several types of cascade datasets. Thus, recent research in predicting the dynamics of diffusion cascades has trended toward developing deep neural network models that can achieve high prediction accuracy [4].

Xu et al. [5] proposed a novel framework for deep-learning-based cascade dynamics prediction tasks called Contrastive Cascade Graph Learning (CCGL). CCGL was inspired by the success of pre-trained models in the domains of natural language processing and computer vision [5]. In these domains, pre-trained models are constructed using large amounts of labeled and unlabeled data. The pre-trained models are then fine-tuned for a specific task and dataset, allowing them to achieve high accuracy. CCGL adopts a similar pre-training and fine-tuning paradigm. It learns generic representations of information cascades through pre-training with labeled and unlabeled datasets of information cascades, then fine-tunes the pre-trained model for a specific prediction task and dataset. The pre-trained models constructed with CCGL are not specific to any particular prediction task and can be applied to several tasks involving information diffusion cascades. It is also expected that these models will contribute to constructing robust prediction models by leveraging the knowledge acquired during pre-training, particularly when the dataset to which they are applied has limited training data.

However, the effectiveness of CCGL has not been fully evaluated yet. Firstly, the transferability of CCGL across different domains has been still unclear. While the transferability within social media datasets (e.g., Twitter and Weibo) was examined in [5], the transferability across different domains, such as from social media to paper-citation datasets, has not been explored. Understanding the transferability of pre-trained CCGL models is crucial for practical application. Secondly the potential utility of model-generated synthetic cascade data for training CCGL models has not been investigated. Despite the anticipated utility of data augmentation in the prediction of cascade popularity [30, 31], the utilization of model-generated synthetic cascade data within the framework of CCGL has not been explored.

In this study, our primary aim is to achieve a comprehensive understanding of the efficacy of CCGL for cascade popularity prediction, and we address the following three research questions.

(**RQ1**) How does the effectiveness of CCGL vary depending on the combination of the source dataset for pre-training and the target dataset for prediction?
(**RQ2**) How effective are synthetic cascades in CCGL *pre-training*?
(**RQ3**) How effective are synthetic cascades in CCGL *fine-tuning*?

**Fig 1:**
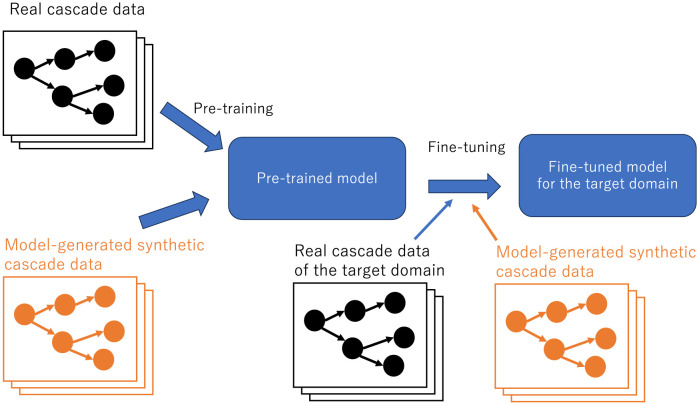
The overview of the learning framework using synthetic cascade data. In the original CCGL framework, real datasets are employed for constructing the pre-trained model and a real dataset from the target domain is used for fine-tuning (depicted in black in the figure). However, in this study, we also use synthetic cascade data for pre-training and fine-tuning (highlighted in orange in the figure). We explore the efficacy of CCGL when pre-training is conducted using synthetic cascade data instead of real data **(RQ2)**. Furthermore, we investigate the effectiveness of CCGL when synthetic cascade data are utilized for fine-tuning in conjunction with real data **(RQ3)**.

We begin by examining the applicability of the CCGL’s pre-trained models to different datasets with different characteristics, allowing us to determine which datasets are suitable for CCGL pre-training **(RQ1)**. Additionally, we examine the feasibility of using synthetic cascade data generated from information diffusion models [20, 21] to build robust prediction models. The inclusion of synthetic cascade data has the potential to significantly augment the training dataset’s size. Consequently, we assess the effectiveness of CCGL when pre-training with synthetic cascade data instead of real data **(RQ2)**. Additionally, we examine the effectiveness of CCGL when synthetic cascade data are incorporated for fine-tuning alongside real data **(RQ3)**. Fig 1 illustrates the overview of the learning framework using synthetic cascade data.

To address the research questions listed above, we examined the effectiveness of CCGL for the cascade popularity prediction task, which uses real and synthetic datasets to predict the future popularity of a given cascade. We used 13 real datasets and six synthetic datasets of information diffusion cascades from experiments on cascade popularity prediction. The real datasets encompassed diverse domains, including social media, paper citation, and question and answer data, varying in size. On the other hand, the synthetic datasets were generated through simulations of information diffusion on synthetic networks derived from network generation models. Through the utilization of these datasets, we constructed CCGL pre-trained models and fine-tuned them using labeled data from each real dataset, enabling us to develop models capable of predicting the future popularity of cascades. By evaluating the prediction accuracy of the resulting models, we can assess their applicability to different datasets, as well as the usefulness of the synthetic cascades.

The main contributions of this paper are as follows.

- We evaluate the effectiveness of CCGL using datasets from various domains and of different sizes. These datasets allow us to assess the transferability of the CCGL models across different datasets.
- We determine the usefulness of model-generated synthetic cascade data for CCGL pre-training. Our results suggest the possibility of constructing robust pre-trained models using synthetic cascade data rather than real cascade data.
- We demonstrate how effective using synthetic cascade data is at improving the prediction accuracy of CCGL and several other popularity prediction models. The experimental results show that synthetic cascade data may be useful for improving the accuracy of not only CCGL, but also other popularity prediction models.

The rest of the paper is organized as follows. In Section Related Work, we give an overview of existing studies on cascade popularity prediction. In Section Preliminaries, we formulate the problem and provide basic definitions. Section Experimental Methodology is an outline of our experimental methodology. In Section Results, we present our results and discuss their implications. Finally, Section Conclusion concludes this paper and discusses future work.

## Related work

Early studies on the cascade popularity prediction problem primarily employed feature-based methods [17, 22]. These methods involved the use of hand-crafted features to build supervised machine learning models for predicting cascade popularity. The accuracy of these prediction models relied heavily on the effectiveness of the selected features, leading researchers to explore the most useful features for predicting cascade popularity. Szabo et al. [17] proposed a cascade popularity prediction method that used temporal features in information diffusion cascades [17]. Jamal et al. [22] proposed a popularity prediction method that used decision tree classifiers [23] and support vector machines [24] based on features such as textual characteristics and community structure of users involved in cascades. While feature-based methods offer interpretability, their performance heavily depends on the quality of the chosen features.

Alternatively, model-based methods based on generative models of information diffusion have been introduced [25–27]. These methods aim to construct interpretable prediction models by learning model parameters from cascade data [25–27]. Based on the idea of survival analysis, Lee et al. [25, 26] proposed a cascade popularity prediction method that uses a Cox proportional hazards regression model [28]. Zaman et al. [27] proposed a stochastic model of the information diffusion cascade over time using Bayesian estimation. While these approaches are useful for predicting the popularity dynamics over time, they are not robust against outliers [29].

Recently, there has been a growing trend in popularity prediction methods towards the use of deep learning, distinguishing them from the aforementioned approaches [4]. Xu et al. [13] proposed a neural network model for popularity prediction that learns latent representations of both structural and temporal features of cascades from the initial sequences of those cascades. Chen et al. [14] proposed using a multi-scale graph capsule network and an influence-attention mechanism for deep-learning-based popularity prediction. Although these deep learning methods have shown promising results compared to feature-based and model-based methods, they often require a substantial amount of labeled data for training the prediction models.

CCGL [5], which is the focus of this paper, is a pioneering method that can be applied to a variety of tasks involved in predicting the future dynamics of information diffusion cascades. CCGL functions by first constructing a pre-trained model that uses a self-supervised contrastive learning framework to learn generic representations of cascades using both labeled and unlabeled cascade data. The pre-trained model is then fine-tuned on a specific target dataset and a specific task. Xu et al. [5] evaluated the transferability of the pre-trained models of CCGL using two social media datasets, Weibo and Twitter. In contrast, the pre-trained CCGL models are expected to be transferable to datasets on platforms other than Twitter and Weibo, to datasets of languages other than English and Chinese, and even to datasets in other domains outside of social media. Therefore, in this paper, we perform a comprehensive evaluation of the transferability of models constructed by CCGL using various datasets. Furthermore, we evaluate the effectiveness of models trained on synthetic cascade data, as well as those trained on real data.

Although research into strategies for addressing the challenge of insufficient labeled data in deep learning-based cascade popularity prediction is still in its early stages, data augmentation is considered to be a promising technique. Hassani et al. [30] proposed methods for extending cascade data through operations such as masking nodes and adding and removing connectivity in real cascade graphs. Zhao et al. [31] proposed a method for extending cascade data using a graph autoencoder. These studies generated synthetic cascade data from real cascade data and used the generated synthetic cascades for model training. In this paper, however, we generate synthetic cascades by using information diffusion models without real cascade data and examine the effectiveness of such synthetic cascade data in training cascade prediction models.

## Preliminaries

### Definitions, problem formulation, and evaluation metric

In this section, we provide definitions of the terms and symbols used throughout the paper, as well as a formulation of the cascade popularity prediction problem. Additionally, we define the evaluation metric used in our experiments. Table 1 shows the definitions of the symbols used in this paper.

**Table 1:** Definitions of the symbols used in this paper.

Symbol	Description
*G*_*i*_, ${\tilde{G}}_{i}$	Cascade graph and augmented cascade graph.
*V*_*i*_, *E*_*i*_	Node and link sets in cascade graph *G*_*i*_.
*i*	Information item.
*C*_*i*_	Cascade for information item *i*.
$u_{j}^{i}$	The *j*th user involved in cascade *C*_*i*_.
$t_{j}^{i}$	The time when user $u_{j}^{i}$ is involved in cascade *C*_*i*_.
*t*_*o*_	Observation time of the cascade.
*t*_*p*_	Prediction time of the cascade.
*P*_*i*_(*t*_*p*_)	Popularity of cascade *C*_*i*_ at time *t*_*p*_.
*M*	Number of nodes in the cascade graph
*N*	Number of labeled cascades

Following the definition given in [5], we define the information cascade graph as follows.

**Definition 1** (**Information cascade graph** [5]) *In social media, an “information cascade” refers to a sequence of individuals (i.e., users) who post and spread information. For instance, a sequence of users who tweet and retweet a particular message on Twitter represents an information cascade. Suppose that an information item i is disseminated M times until time t. Let u*_*k*_ *be the k-th user who disseminated information item i*, (*u*_*j*_, *u*_*k*_) *be the k-th diffusion path, and t*_*k*_ *be the time of the k-th diffusion. Note that* (*u*_*j*_, *u*_*k*_) *represents that item i is transmitted from user u*_*j*_ *to user u*_*k*_. *Then, the information cascade C*_*i*_(*t*) *of item i at time t is defined as follows*:

$$\begin{array}{c} C_{i}\left(t\right)=\left\{\left(u_{k},t_{k}\right)|k\in \left[1,M\right],t_{k}<t\right\}. \end{array}$$ (1)

*Additionally, the information cascade graph of information item i is denoted as G*_*i*_(*t*) = (*V*_*i*_, *E*_*i*_), *where* *V*_*i*_ = {*u*_*k*_|1 ≤ *k* ≤ *M*} *is the set of users in cascade C*_*i*_(*t*), *and E*_*i*_ = {(*u*_*j*_, *u*_*k*_)|1 ≤ *j*, *k* ≤ *M*} *is the set of all diffusion paths for the information item i*.

With this definition, the cascade popularity prediction problem can be formulated as follows [5].

**Problem 1** (**Cascade popularity prediction** [5]) *Let G*_*i*_(*t*_*o*_) *be the cascade graph of item i at observation time t*_*o*_. *The goal is to predict the popularity P*_*i*_(*t*_*p*_) *of information item i at a specified prediction time t*_*p*_ (*where t*_*p*_ ≫ *t*_*o*_).

Following existing studies [5, 14], we use the mean squared logarithmic error (MSLE) [32] as a metric for evaluating the accuracy of a cascade popularity prediction. Let *N* be the number of information cascades for a prediction, then MSLE is defined as

$$\begin{array}{c} \text{MSLE}=\frac{1}{N}\sum_{i=1}^{N}{\left(\text{log}\;{\hat{P}}_{i}\left(t_{p}\right)-\text{log}\;P_{i}\left(t_{p}\right)\right)}^{2}, \end{array}$$ (2)

where *P*_*i*_(*t*_*p*_) represents the true popularity of information *i*, while ${\hat{P}}_{i}\left(t_{p}\right)$ denotes the predicted popularity of information *i* at time *t*_*p*_. Lower MSLE scores correspond to higher prediction accuracy.

### Overview of CCGL

In this section, we present an overview of the basic concepts behind CCGL for cascade popularity prediction. For a more comprehensive understanding of CCGL, we recommend referring to the original paper by Xu et al. [5].

CCGL employs self-supervised learning techniques to construct a model capable of predicting the future popularity *P*_*i*_(*t*_*p*_) of an information item *i* within a given cascade graph *G*_*i*_(*t*_*o*_) at observation time *t*_*o*_, utilizing both labeled and unlabeled cascade data. A labeled cascade consists of a cascade graph *G*_*i*_(*t*_*o*_) and its corresponding future popularity *P*_*i*_(*t*_*p*_), whereas an unlabeled cascade contains solely the cascade graph *G*_*i*_(*t*). A pre-trained model is constructed using both labeled and unlabeled data, and it is further fine-tuned using the labeled data specific to the target dataset for which the prediction is being performed.

During this process, an augmented cascade graph ${\tilde{G}}_{i}$ is created for each information item *i* and utilized for model training. The augmented cascade graph ${\tilde{G}}_{i}$ is obtained by introducing node additions and deletions within the original cascade graph *G*_*i*_(*t*_*o*_). The addition and deletion of nodes simulate the process of information diffusion. The attractiveness of each node, proportional to its degree in the original cascade graph, is calculated. Newly added nodes are connected to existing nodes with a probability corresponding to their attractiveness. To maintain graph connectivity, when deleting a node, the link connected to the leaf node is removed. The node to be deleted is selected based on the probability proportional to its parent node’s degree.

To obtain generic representations of cascade graphs in CCGL, a contrastive learning framework is used to encode the cascade graph. More specifically, the graph encoder that was also used in [33] is used. The graph encoder has two main components: (i) graph embedding based on spectral graph wavelets and (ii) an interactive GRU-based network for learning contextual user behavior in cascade data [5]. These components map the cascade graph *G*_*i*_ to a fixed-length representation $h_{i}\in R^{d_{h}}$. Subsequently, a multilayer perceptron (MLP)-based projection head is employed to project ***h***_*i*_ onto a new representation $z_{i}\in R^{d_{z}}$. The representation ***z***_*i*_ is utilized for computing the contrastive loss and optimizing the CCGL framework.

### Information diffusion models

To address RQ2 and RQ3, we use two information diffusion models for generating synthetic cascade data, the independent cascade (IC) model [20] and the Profile model [21]. The IC model is driven by the sender of the information and is often used as a model for information diffusion on social media. The Profile model assumes that the information diffusion process depends on the interests of the nodes that receive the information.

We assume that the social network is represented by a directed network *H* = (*V*, *E*), where *V* is the set of all nodes and *E*(⊂ *V* × *V*) is the set of all links. In both models, a node is referred to as “active” if it receives information. The information diffusion process is assumed to start from an initial active node and proceed in discrete time *t* ≥ 0, with the node state changing from inactive to active but not vice versa. The IC and Profile models terminate when no more attempts to activate any inactive nodes can be made.

## Experimental methodology

### Experimental settings

The observation time *t*_*o*_ and prediction time *t*_*p*_ are determined for each dataset, then each information item *i* is classified as either labeled or unlabeled data. Table 2 provides an overview of our real datasets, including the amounts of labeled and unlabeled data, as well as the duration of the observation and prediction periods based on the specified times. More detailed information on dataset construction can be found in the S1 Appendix. For each information item *i* classified as labeled data, a cascade graph *G*_*i*_(*t*_*o*_) is constructed at the observation time *t*_*o*_, and the future popularity *P*_*i*_(*t*_*p*_) at the prediction time *t*_*p*_ is obtained. Therefore, for a labeled cascade *i*, a pair *G*_*i*_(*t*_*o*_) and *P*_*i*_(*t*_*p*_) is available. For each information item *i* classified as unlabeled data, a cascade graph *G*_*i*_(*t*_*p*_) is constructed at the prediction time *t*_*p*_. Only *G*_*i*_(*t*_*p*_) is available for the unlabeled cascade *i*. Labeled data are used for training and testing, while unlabeled data are used only for pre-training. Consistent with Xu et al. [5], cascade graphs with fewer than 10 nodes are excluded from our analysis. Additionally, for cascade graphs containing more than 100 nodes, we selected the first 100 nodes (arranged by their adoption time) to construct the cascade graphs.

**Table 2:** Overview of the datasets.

Datasets	Platform	Language	Diffusion mechanism	Num. of labeled cascades	Num. of unlabeled cascades	Num. of nodes	Avg. observed num. of nodes	Avg. path length	Prediction time	Observation time
Virality [34]	Twitter	English	Retweet	18,187	7,412	206,772	31.03	84.21	2 days	32 days
Nepal [35]	Twitter	Nepali	Retweet	4,586	1,889	110,487	31.43	47.63	2 hours	2 days
withURL [36]	Twitter	English	Retweet	890	314	9,187	37.42	44.74	1 day	11 days
DeepHawkes [19]	Weibo	Chinese	Retweet	39,076	19,431	1,301,201	39.88	98.13	1 hour	1 day
DiffuGreedy [37]	Weibo	Chinese	Retweet	91,018	44,633	91,018	60.94	189.01	2 months	39 months
SSRDGAN [38]	Douban	Chinese	Review	6,257	2,212	23,495	29.19	44.26	1 year	12 years
ComSoc [39]	Douban	Chinese	Review	3,608	6,254	16,941	27.65	38.98	6 months	6 years
Digg [40]	Digg	English	Like	1,593	97	26,853	99.94	769.26	2 days	32 days
Android [41]	Stack-Exchanges	English	Comment	35	43	1,979	25.37	25.37	6 months	7 years
Christianity [41]	Stack-Exchanges	English	Comment	23	24	964	20.51	20.51	6 months	6 years
ACM [42]	ACM	-	Cite	12,988	20,013	20,6071	12.57	12.59	3 years	10 years
APS [5]	APS	-	Cite	27,802	44,921	325,675	12.38	17.72	3 years	20 years
DBLP [42]	DBLP	-	Cite	1,736	44	6,832	17.70	17.77	5 years	20 years

50% of the labeled data were randomly selected as training data, 10% as validation data, and 40% as test data. When pre-training CCGL on a dataset, we use the training and unlabeled data for that dataset. When applying a pre-trained model to a target dataset, we fine-tune it using the training and validation data for that dataset, then apply the fine-tuned model to the test data for that dataset to obtain the prediction accuracy. In our experiments, we used the same parameter settings as those in [5], where CCGL was proposed.

### Datasets

We used 13 public datasets of real information cascades, which are given in Table 2. These datasets differ in terms of the social media platforms, languages, and mechanisms used for information diffusion. Some of these datasets also contain information on social networks, the basic statistics of which are given in Table 3. The social networks were used to generate synthetic cascade data for fine-tuning, as described in Section Synthetic cascade generation for fine-tuning. We used several datasets with different characteristics so that we could evaluate how well CCGL works across different datasets.

**Table 3:** Statistics of social networks.

Datasets	Num. of users	Num. of links
Virality	595,460	14,273,311
Nepal	273,222	17,819,610
DiffuGreedy	1,776,950	308,489,739
withURL	12,627	619,262
SSRDGAN	695,800	1,758,302
ComSoc	959,289	5,130,107
Digg	279,631	1,731,658
Android	9,942	48,574
Christianity	2,899	35,625

In addition to the real datasets, we also utilized synthetic datasets generated through simulations of information diffusion models. The procedure for generating synthetic cascades is as follows. A graph *G* is generated using a network generation model. At time *t* = 0, one randomly selected node on *G* is activated as a seed node, and a simulation of information diffusion from the seed node is run based on either the IC model [20] or the Profile model [21]. The sequence of nodes and their corresponding timestamps when they become active during each simulation run *i* are considered as a diffusion cascade of information item *i*.

In this study, we generated six synthetic cascade datasets by combining three network generation models and two information diffusion models. The three network generation models were the Barab’asi-Albert (BA) model [43], the Lancichinetti-Fortunato-Radicchi Benchmark model (LFR model) [44], and the Watts-Strogatz (WS) model [45]. The two information diffusion models were the IC model [20] and the Profile model [21]. The diffusion probabilities in the IC and Profile models were determined based on the weighted cascade (WC) model [46]. Specifically, the information diffusion probability from node *u* to node *v* in the IC model is defined as $p_{u,v}=1/d_{v}^{\text{in}}$, where $d_{v}^{\text{in}}$ is indegree of node *v*. The link weight on link (*u*, *v*) in the Profile model was defined as $q_{u,v}=1/d_{v}^{\text{in}}$. The number of nodes in the three synthetic networks was set to *N* = 10000, the number of additional links in the BA model to *m* = 10, the average degree in the WS model to *k* = 10, and the probability of choosing an interconnected link to *p* = 0.01. In the LFR model, the power-law exponent of the degree distribution was set to *τ*_1_ = 2.5, the power-law exponent of the community size distribution to *τ*_2_ = 1.5, the mixing parameter to *μ* = 0.1, the mean degree to *k* = 10, and the maximum degree to *k*_max_ = 100. The information diffusion model was simulated up to a maximum of *t* = 10 and generated 5000 information diffusion cascades with a diffusion size of more than 10. Similarly to real datasets, the prediction time for synthetic datasets was *t*_*p*_ = 10, and the observation time was *t*_*o*_ = 3.

### Synthetic cascade generation for fine-tuning

To evaluate the effectiveness of synthetic cascade data for fine-tuning, we generated synthetic cascades by running simulations of an information diffusion model on the social networks for each real dataset. For each real dataset, we generated the same number of synthetic cascades as the number of training cascades in the dataset. The simulation parameters were the same as those described in the previous section. In order to ensure consistency in the diffusion time between the synthetic and real cascades within each dataset, we transformed the diffusion time *t* of the synthetic cascades. For each discrete simulation time *t* in a synthetic cascade, we randomly assigned a diffusion time selected from the real cascades. More specifically, the procedure was as follows. First, all diffusion times in the real dataset were listed in ascending order. Then, we assigned all of the diffusion times to 10 equally divided sections. Each discrete timestamp *t* = *i*(1 ≤ *i* ≤ 10) in a synthetic cascade was replaced with a real diffusion time randomly selected from the *i*-th section. The resulting synthetic cascades, along with the real cascades in each dataset, were then used for fine-tuning the model. This allowed us to leverage both real and synthetic cascade data to improve the prediction performance.

### Baselines

In addition to the CCGL, we used the following methods to perform the cascade popularity prediction.

- **Base** [5]: The Base model is a baseline model for popularity prediction used in [5]. It is a standard deep-learning model that consists of a cascade graph encoder and a multilayer perceptron-based projection head.
- **CasFlow** [13]: CasFlow is a model for predicting the popularity of information cascades by learning latent representations of both structural and temporal information. It uses a hierarchical variational information diffusion model to learn the posterior distribution of the cascade distribution using variational inference and normalized flows. CasFlow is a state-of-the-art deep-learning-based model for cascade popularity prediction.
- **MUCas** [14]: MUCas (Multi-scale Graph Capsule Network for Popularity Prediction of Information Cascades) is another state-of-the-art model for deep-learning-based cascade popularity prediction. It decomposes the observed cascade graph into a series of subcascade graphs based on discrete time intervals using a time-interval-aware subcascade graph sampling method. The model then uses a multiscale graph capsule network and an influence-attention mechanism to learn the cascade representation and predict its popularity.
- **CasCN** [47]: CasCN (Recurrent Cascades Convolutional Networks) is another benchmark model for deep-learning-based cascade popularity prediction. Similarly to CasFlow, it aims to model and predict cascades by learning the latent representation of both structural and temporal information. However, CasCN focuses solely on the local structure of cascades, while disregarding the global user behavior.

The hyperparameters for each method, including CCGL, were set to the same values as those used in the original paper.

## Results

### Transferability of CCGL pre-trained models

**Fig 2:**
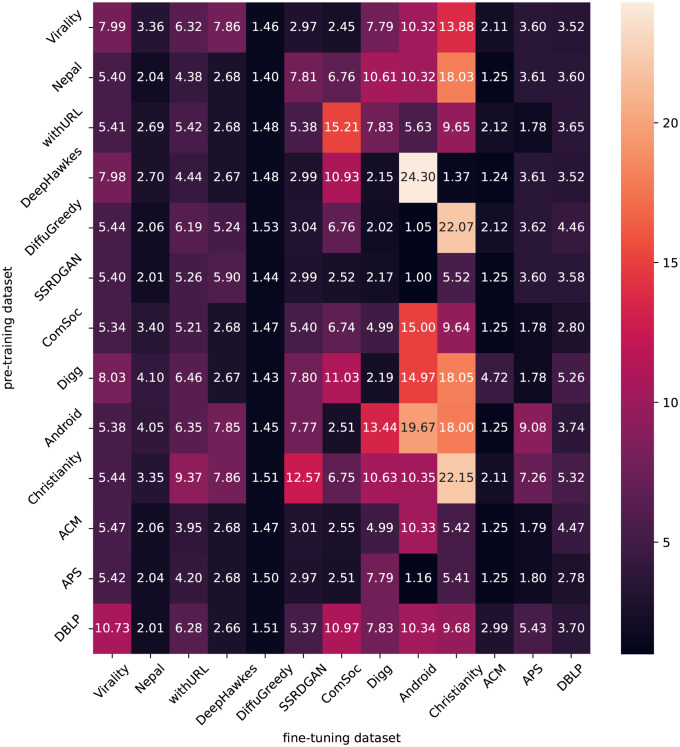
MSLE for combinations of the source dataset used for pre-training and the target dataset used for prediction. The numbers indicate the MSLE values for each combination of pre-training and target datasets. In the case of the Android and Christianity datasets, pre-training on the other datasets led to lower MSLE scores compared to pre-training on these datasets alone.

To address RQ1, we evaluated the prediction accuracy of CCGL for each combination of source dataset used for pre-training and target dataset used for prediction. We constructed a pre-trained model of CCGL for each dataset, then fine-tuned the model using the training and validation data for each target dataset. We then evaluated the prediction accuracy of the fine-tuned model using test data in the target dataset. Specifically, we conducted ten experiments for each combination of the source and target datasets to obtain the prediction accuracy, the results of which are presented in Fig 2.

From Fig 2, we can see that the use of pre-trained models constructed on other datasets is particularly beneficial when the prediction models are applied to datasets with small amounts of training data for fine-tuning. For instance, the Android and Christianity datasets have a very small number of cascades available for training (see Table 2), resulting in extremely high MSLE values of 19.67 and 22.15, respectively (indicating low prediction accuracy), when the models are pre-trained on these datasets. In contrast, when a pre-trained model built on a dataset with a larger number of cascades, such as APS, is applied to a dataset with a smaller amount of training data, such as Android and Christianity, the prediction accuracy is higher than when training only on these datasets. These results suggest that pre-training on other datasets can be effective, especially when the amount of training data in the target dataset is limited. On the other hand, for datasets with a certain amount of training data, there is almost no difference in prediction accuracy between pre-training and fine-tuning on the target dataset alone and pre-training on other datasets while fine-tuning on the target dataset. This suggests that pre-trained models constructed on datasets of a certain size have the potential to be applied to a variety of datasets. Among the datasets used in this experiment, the APS dataset and the SSRDGAN dataset are thought to be particularly suitable as pre-training datasets.

Additionally, Fig 2 suggests that the *similarity* between the pre-training and the target datasets does not have large effects on the prediction accuracy of the obtained model. For instance, there is no significant difference in accuracy when transferring between datasets with the same language and when transferring between datasets with different languages. Similarly, comparing the prediction accuracy of transferring the model from a social media dataset to another social media dataset and transferring the model from a social media dataset to a paper-citation dataset, significant differences cannot be observed. This suggests that the pre-trained models of CCGL have obtained a generic representation of information diffusion cascades, and that differences between the source and target domains have a minimal impact on prediction accuracy.

### Effectiveness of pre-trained models trained on synthetic cascades

**Fig 3:**
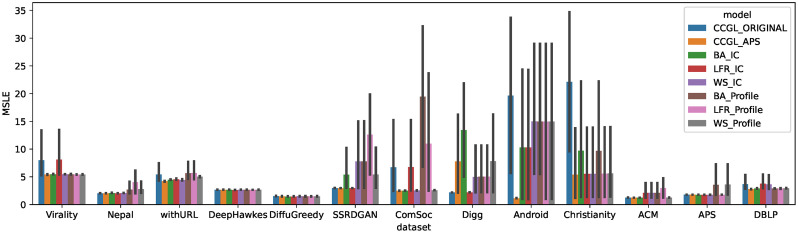
Average MSLE for each combination of synthetic dataset used for pre-training and real target dataset used for prediction. We compare models pre-trained on six distinct synthetic datasets (BA_IC, LFR_IC, WS_IC, BA_Profile, LFR_Profile, and WS_Profile), the model pre-trained on APS dataset (CCGL_APS), and the model pre-trained on the target dataset (CCGL_ORIGINAL). The models pre-trained on the BA_IC and LFR_IC datasets achieve lower MSLE values compared to the CCGL_ORIGINAL model.

We next investigate the effectiveness of pre-trained models trained on synthetic cascade datasets by addressing RQ2. The results in the previous subsection suggest that it is possible to construct a generalizable pre-trained model using any dataset with a certain number of cascades. This allows for the possibility of constructing a pre-trained model that can be applied to the prediction task for each dataset using synthetic cascade data generated from information diffusion models rather than real cascade data. Fig 3 shows a comparison of the prediction accuracies of models pre-trained on six synthetic cascade datasets when they were applied to each real dataset. For comparison purposes, the accuracies of the model pre-trained on the APS dataset (CCGL_APS) and the model pre-trained on each target dataset (CCGL_ORIGINAL) are also shown in the figure.

From Fig 3, we observe that the pre-trained models built on synthetic cascade data demonstrate comparable effectiveness to the pre-trained models built on real data for predicting cascade popularity. Specifically, when applied to datasets with a small amount of training data, such as Android and Christianity, the models pre-trained on the BA_IC and LFR_IC datasets achieve higher prediction accuracy than those pre-trained on the target dataset alone. Furthermore, the prediction accuracies of the models pre-trained on the BA_IC and LFR_IC datasets are comparable to those of the models pre-trained on the APS dataset for many datasets. This indicates that pre-training on a synthetic dataset can yield a robust pre-training model applicable to various other datasets. Among the models examined in this study, BA_IC and LFR_IC are particularly suitable for pre-training. Given that the primary goal of pre-training in CCGL is to obtain a generic representation of the cascade graphs, synthetic cascades are deemed valuable for this purpose. This finding is particularly useful in scenarios where only limited real data is available. The results suggest that when generating synthetic cascades, it is effective to use the BA or LFR network as the network and the IC model as the information diffusion model. This is thought to be because these settings generated data close to the real cascade.

### Effectiveness of synthetic cascades for fine-tuning

Next, we examine the effectiveness of synthetic cascade data for fine-tuning (RQ3). The results from the previous subsection suggest that it will be effective when added to the training data together with the real data.

**Fig 4:**
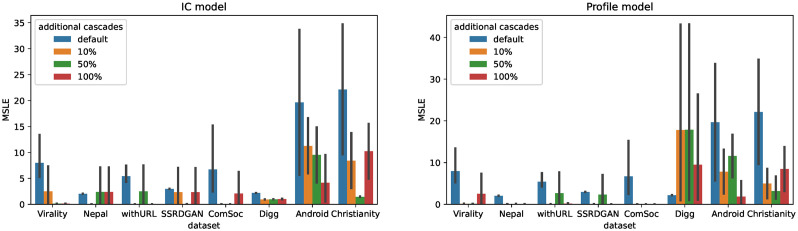
Average MSLE when using additional synthetic cascades for fine-tuning. We compare models under varying amounts of additional synthetic cascade data. The default MSLE scores of models without the incorporation of additional synthetic cascade data are also shown. The MSLE scores obtained when using additional synthetic cascade data are lower than the MSLE scores when only using real cascade data.

We built a pre-trained model of CCGL on each target dataset and fine-tuned it using the training and validation data in the target dataset and the generated synthetic cascade data, as explained in Section Synthetic cascade generation for fine-tuning. The amount of additional synthetic cascade data used for fine-tuning was [10%, 50%, and 100%] of the amount of training data. The prediction accuracy of the fine-tuned model was evaluated using test data from the target dataset. Fig 4 shows a comparison of the prediction accuracy when fine-tuning is performed with additional synthetic cascade data. Note that datasets without social network data were excluded in this experiment because social network data were necessary for generating synthetic cascades. Additionally, the DiffuGreedy dataset was also excluded owing to memory constraints on the machine used for the experiments.

From Fig 4, it is evident that fine-tuning with additional synthetic cascades significantly improves the prediction accuracy for all datasets. The improvement is particularly prominent when the amount of additional synthetic cascades is 10% or 50% of the real data, and when the IC model is used. For example, when employing the IC model, the t-test reveals that the MSLE scores achieved by incorporating an additional 50% of synthetic cascades are significantly lower than the MSLE scores of the default models (i.e., the models excluding any additional synthetic cascade) on the Virality, SSRDGAN, Digg, and Christianity datasets (*p* < 0.05). These results indicate that the synthetic cascades generated from the IC model can serve as valuable additional training data for fine-tuning, leading to improved prediction accuracy of the fine-tuned models. The Christianity dataset, exhibiting particularly substantial improvements, has a significantly low number of labeled cascades (Table 2). Consequently, conventional fine-tuning approaches would have exhibited suboptimal performance given the scarcity of labeled authentic data. Conversely, through the introduction of synthetic cascades, we achieved noteworthy enhancements in prediction accuracy. This holds true even for datasets containing a certain volume of labeled data. This finding highlights the effectiveness of incorporating synthetic cascades in the training process, particularly when using the IC model. However, the extent of this improvement in prediction accuracy varies depending on the dataset. Further study is needed to clarify in what datasets the additional synthetic cascade would be useful.

### Comparison with baselines

**Fig 5:**
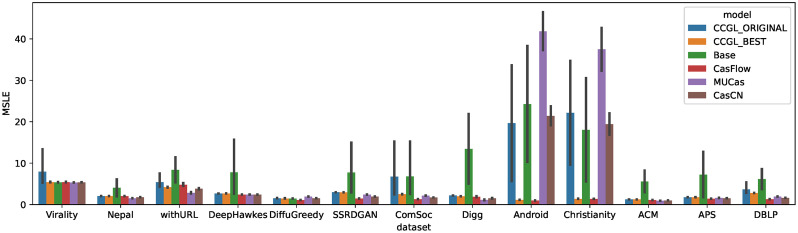
Comparison of MSLE among CCGL and the baselines. The MSLE of CCGL_BEST is lower than or comparable with other baselines. The MSLE of CCGL_ORIGINAL is lower than or comparable to Base, MUCAS, and CasCN, but higher than CasFlow.

Finally, we compared the prediction accuracies of the CCGL models to those of other methods. While CCGL is a method that is not specific to a particular task, a number of other prediction methods have been proposed that specialize specifically in the cascade popularity prediction task. We can assess the effectiveness of CCGL by comparing it to other state-of-the-art methods for cascade popularity prediction. Fig 5 presents a comparison of the prediction accuracies of CCGL and the baseline methods on each dataset. CCGL_ORIGINAL shows the results of building a pre-trained model on each dataset and fine-tuning them on each target dataset, and CCGL_BEST shows the results from using the pre-trained model with the best prediction accuracy for each target dataset.

First, we compare CCGL_ORIGINAL to other methods. Note that CCGL and the other baselines are all trained on the same amount of data. Fig 5 shows that CCGL demonstrates higher prediction accuracy than the Base and MUCas models when trained on the same amount of data. The differences in accuracy are statistically significant (*p* < 0.05). However, CasFlow, a state-of-the-art method, achieves slightly better prediction accuracy than CCGL_ORIGINAL, and the differences are also statistically significant (*p* < 0.05).

Next, we compare CCGL_BEST with the other methods. From Fig 5, we can see that the prediction accuracies of CCGL_BEST and CasFlow are comparable. This suggests that simply pre-training on the target dataset does not necessarily guarantee good prediction accuracy for CCGL. To construct an accurate prediction model using CCGL, it is important that it be pre-trained on a large dataset, especially when it is then applied to a dataset with a small amount of training data. We should also note that CasFlow achieves generally higher prediction accuracy than CCGL_BEST. Except for the Android and Christianity datasets, the differences of MSLE between CCGL_BEST and CasFlow are significant (*p* < 0.05).

In our experiments, CasFlow achieved high accuracy in all datasets, suggesting that further study is needed to clarify the superiority of CCGL over task-specific methods like CasFlow. For example, the superiority of CCGL may be confirmed in cases where the amount of training data in the target dataset is more limited. Additionally, since CCGL can be used for tasks other than popularity prediction, it will be necessary to evaluate its effectiveness in various other tasks in the future. The original paper that proposed CCGL did not compare it with state-of-the-art methods like CasFlow, but the experiments in this paper make CCGL’s performance more clear. We believe that further development of contrastive self-supervised learning methods will be needed in the future for various applications utilizing cascade data.

**Fig 6:**
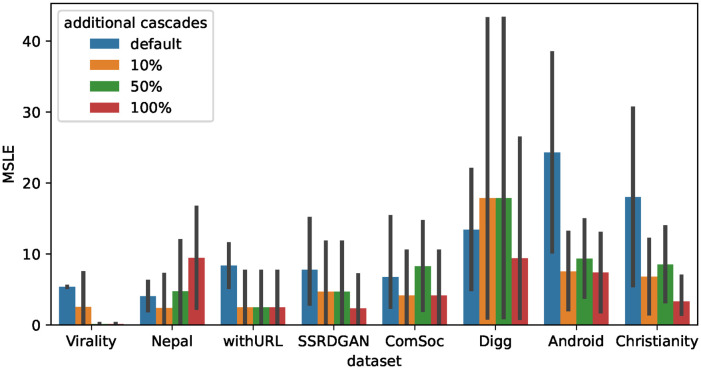
Average MSLE when using additional synthetic cascades generated from the IC model to train Base. Using additional synthetic cascades generally improves the MSLE scores of the Base.

**Fig 7:**
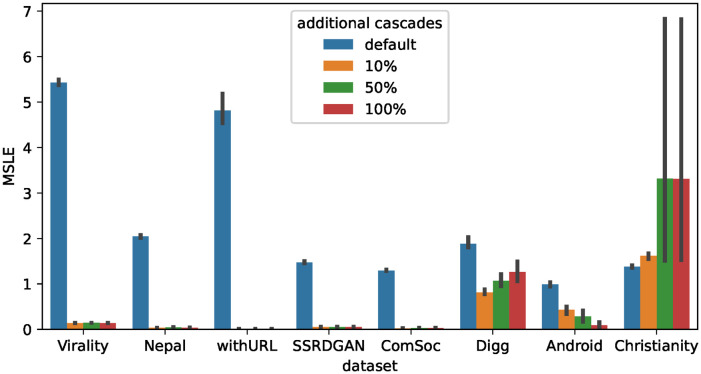
Average MSLE when using additional synthetic cascades generated from the IC model to train CasFlow. Using additional synthetic cascades generally improves the MSLE scores of the CasFlow.

**Fig 8:**
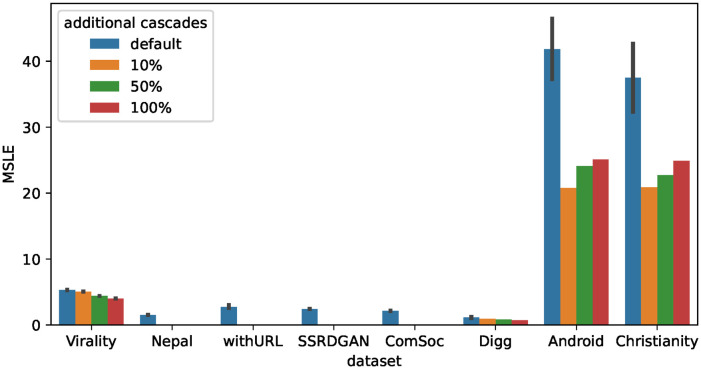
Average MSLE when using additional synthetic cascades generated from the IC model to train MUCas. Using additional synthetic cascades generally improves the MSLE scores of the MUCas.

Next, we examine the effectiveness of synthetic cascade data for baseline methods. Figs 6–8 show the accuracy of the prediction models trained with real data and additional synthetic cascades for Base, CasFlow, and MUCas. Note that the results in Section Effectiveness of synthetic cascades for fine-tuning suggest that the IC model will be effective, so we only show the results for synthetic cascades generated by the IC model.

From Figs 6–8, we can see that on most datasets using additional synthetic cascades for training significantly improves the prediction accuracy of the baseline methods. The only exceptions are the Digg dataset of Base and the Christianity dataset of CasFlow. Similar to the results in Section Effectiveness of synthetic cascades for fine-tuning, the prediction accuracy was greatly improved when using an additional 10% or 50% of synthetic cascades. This suggests that for cascade popularity prediction methods, synthetic cascades generated from the IC model can be used as training data to improve the accuracy of the prediction models.

## Conclusion

In this paper, we have conducted an extensive evaluation of the transferability of pre-trained models of CCGL [5] for popularity prediction using a diverse set of real and synthetic datasets. While a previous study [5] focused on evaluating transferability using two social media datasets, our work expands on this by considering multiple datasets from different domains, languages, social media platforms, and diffusion time scales. The results demonstrate that the pre-trained CCGL models exhibit strong transferability across various real datasets. Moreover, we have shown that the pre-training and fine-tuning framework of CCGL is particularly effective when the training data in the target domain is severely limited.

One of the key findings of this paper is the effectiveness of synthetic cascade data in both pre-training and fine-tuning. The use of synthetic cascades generated from information diffusion models to build robust prediction models has not been explored previously. Our results show that the MSLE scores of models pre-trained on real data and those pre-trained on synthetic data are comparable, indicating that synthetic cascades alone can be used to construct a robust pre-trained model. Furthermore, we demonstrate that synthetic cascade data is beneficial for fine-tuning CCGL. Models trained using both real and synthetic cascades achieve significantly lower MSLE scores compared to models trained using only real cascades. For instance, the MSLE score of the model trained solely on real cascades on the Virality dataset was 7.99. However, when incorporating an additional 50% of synthetic cascades for training, the MSLE score of the model significantly improved to 0.144. The effectiveness of synthetic cascades is also observed in training other popularity prediction models, such as CasFlow and MUCas. These findings highlight the utility of synthetic cascade data generated from information diffusion models for training popularity prediction models.

We recognize some limitations in this study. First, CCGL can be used for several tasks involving cascade data, but we focused on only the cascade popularity prediction task. To fully understand the effectiveness of CCGL, we need to investigate its transferability across different types of tasks. Second, how the procedures of synthetic cascade generation affect the results is still unclear. Our first step in using synthetic cascades for popularity prediction was to construct several datasets using the IC [20] and Profile [21] models. These models have several parameters, and how these parameters affect the results is unclear. Moreover, the effectiveness of other information diffusion models is also unclear. Ultimately, more efforts are needed to develop effective ways of generating synthetic cascade data.

## Supporting information

S1 Appendix Details of the datasets. (PDF)
