# Supplementary material for: On the effectiveness of a contrastive cascade graph learning framework: The power of synthetic cascade data
Source: PLoS One. 2023 Oct 16;18(10):e0293032. doi: 10.1371/journal.pone.0293032 (PMC10578604; doi:10.1371/journal.pone.0293032)
Supplement: S1 Appendix — (PDF) [file pone.0293032.s001.pdf]

Appendix.

Details of the Datasets Following Xu et al. [1], we determined the observation time and the prediction time for each dataset. The prediction time is the time of the final information diffusion for every dataset. The observation times for the Virality, DeepHawkes, ACM, APS, and DBLP datasets are the same as those used in [1]. For the other datasets, the observation time was defined as the ratio of the length of the observation period to the length of the prediction period, and was similar for all datasets. Here, the length of the prediction (observation) period is the elapsed time from the posting of the earliest information item in the dataset to the prediction (observation) time. The prediction times and the observation times shown in Table **??** are the lengths of the prediction and observation periods.

We classified each information item i as either labeled or unlabeled data. If information item i was posted after the threshold time t_s, then it was classified as unlabeled data, otherwise, it was classified as labeled data. Following Xu et al. [1], the threshold time was defined as $t_s = \frac{t_f + t_p}{2}$, where t_f is the posting time of the earliest posted information item in the dataset.

While the explicit diffusion paths of cascades are available for the Virality, DeepHawkes, ACM, APS, and DBLP datasets, they are not for the other datasets. Therefore, for datasets that do not contain explicit diffusion paths, we obtain diffusion paths of cascades by using the social network $H = (V, E)$ in the dataset. For each information item i, the k-th diffusion path (u_j, u_k) was determined as follows.

1. Extract the set of users who diffused information item i before the current user u_k and denote it as $W = \{u_j | t_j^i < t_k^i\}$.
2. Identify the user u_j with the largest j value among the users $u_j \in W$ that have a social network connection with u_k (i.e., $(u_k, u_j) \in E$). This is the last user among the users followed by u_k who spread information item i before u_k.
3. If such a user u_j exists, then the k-th diffusion path is determined as (u_j, u_k). If not, then the diffusion path is assumed to be from the first user who posted information item i to u_k (i.e., the k-th diffusion path is determined as (u_1, u_k)).

References

1. Xu X, Zhou F, Zhang K, Liu S. CCGL: Contrastive Cascade Graph Learning. IEEE Transactions on Knowledge and Data Engineering. 2022;.
